# Supplementary material for: Evolving Role of [18F]Flurocholine PET/CT in Assessing Primary Hyperparathyroidism: Can It Be Considered the First-Line Functional Imaging Approach?
Source: J Clin Med. 2023 Jan 19;12(3):812. doi: 10.3390/jcm12030812 (PMC9917644; doi:10.3390/jcm12030812)
Supplement: Supplementary file 1 [file jcm-12-00812-s001.zip › jcm-2095118-supplementary.pdf]

Journal of
Clinical Medicine

MDPI

Supplementary Materials

Study Population

Overt primary hyperparathyroidism (oPHPT) was defined as elevated serum levels of both iPTH and calcium. Patients diagnosed with mild PHPT (mPHPT) were: a) NCmPHPT: patients with normal serum calcium levels and elevated serum intact iPTH levels; and b) NHmPHPT: patients with normal serum iPTH levels and elevated serum calcium levels. Based on the utilized laboratory kits, the normal range for serum calcium level was considered 2.15–2.55 mmol/L. iPTH had the normal range of 16–65 pg/mL.

In the oPHPT population, 271 patients who underwent either surgical or conservative approaches were included. In 200 (74%) patients who underwent surgery (n = 200), [^{18}F]FCH PET/CT imaging was performed before the surgery, with an interval of no longer than one month (mean days interval = 21 ± 8). Most patients underwent MIP (96%), and BNE was performed in only 8 (4%) patients. Noteworthy, all MGD patients did not undergo BNE since a few were found to be MGD retrospectively. A conservative treatment approach was applied to the remaining 71 (26%) patients. The most common reasons for choosing the conservative approach were asymptomatic patients, evidence of underlying disease, high risks of surgical complications, or negative initial imaging results in the initial examinations.

In the mPHPT population, 50 patients were included. All underwent [^{18}F]FCH PET/CT imaging within a month of diagnosis (mean days interval = 19 ± 11). A total of 23 (46%) patients were managed with surgical resection (20 and 3 patients underwent MIP or BNE, respectively), and 27 (56%) patients were chosen for the conservative approach. Like the oPHPT group, management consideration was done following a multi-disciplinary decision after reviewing all clinical and imaging findings and underlying thyroid disease.

[^{18}F]FCH PET/CT Acquisition and Interpretation

[^{18}F]FCH PET/CT imaging was performed from the skull base to the diaphragm in a supine position using two PET/CT scanners (Philip Ingenuity TF, Netherlands; Siemens Biograph mCT, Erlangen, Germany). The details varied depending on the scanner, 75 s per bed for Philip's scanner and 100 s per bed for Siemens. Slice thickness of 2mm and matrix size of 288 × 288 were used in both. Approximately 200 MBq of [18F]FCH was injected into each patient. Imaging was performed 60 min after radiotracer injection. A non-contrast-enhanced low-dose CT scan (Biograph mCT with a matrix size of 400 × 400 and slice thickness of 3mm) was performed for attenuation correction and precise anatomical location. Slice measuring 3mm iterative algorithm (Siemens: PSF + TOF, 2 iterations, and 21 subsets and Philips: BLOB-OS-TF) were used for reconstruction in both scanners. Both PET/CT scanners were accredited by EARL/EANM (EANM Research Ltd./European Association of Nuclear Medicine).

Two experienced nuclear medicine physicians aware of the patient's diagnosis evaluated [^{18}F]FCH PET/CT images independently. The scans were analysed using dedicated software (2020 Koninklijke Philips N.V., Philips intelliSpace portal). Any focus of non-physiological [^{18}F]FCH uptake (+/− correlation in the CT scan) in the parathyroid region and upper mediastinum higher than background uptake was considered HFPT.

Supplemental Tables

Table S1. Definition of the [^{18}F] FCH PET/CT findings compared to the reference standard in oPHPT group.

	**Surgical approach**	**Conservative approach**
**True positive**	Patients with positive [^{18}F]FCH PET/CT and histopathological results showing normal or decreased serum levels of iPTH and Ca on follow-up.	Patients with positive [^{18}F] FCH PET/CT revealed stable or increased serum levels of iPTH or Calcium or showed positive follow-up imaging.
**True negative**	Patients with negative [^{18}F]FCH PET/CT imaging and negative histopathology results	Patients with negative [^{18}F]FCH PET/CT imaging that experienced the decline in the level of iPTH or Calcium on clinical follow-ups
**False positive**	Patients who showed positive [^{18}F]FCH PET/CT results but negative histopathology	Patients with positive [^{18}F]FCH PET/CT imaging but showed the decline in serum level of iPTH or Calcium on clinical follow-ups
**False negative**	Patients with negative [^{18}F] FCH PET/CT but positive histopathology.	Patients with negative [^{18}F] FCH PET/CT but positive clinical and imaging follow-ups.

Table S2. Definition of the [^{18}F] FCH PET/CT findings compared to the reference standard in mPHPT group.

	**Surgical approach**	**Conservative approach**
**True positive**	Patients with positive [^{18}F]FCH PET/CT and histopathological results	Patients with positive [^{18}F] FCH PET/CT and revealed stable or increased serum level of iPTH or calcium or showed positive follow up imaging.
**True negative**	Patients with negative [^{18}F]FCH PET/CT imaging and negative histopathology results	Patients with negative [^{18}F]FCH PET/CT imaging that experienced a decline in the level of iPTH or calcium on clinical follow-ups
**False positive**	Patients who showed positive [^{18}F]FCH PET/CT results but negative histopathology	Patients with positive [^{18}F]FCH PET/CT imaging but showed a decline in serum level of iPTH or calcium on clinical follow-ups
**False negative**	Patients with negative [^{18}F] FCH PET/CT but positive histopathology.	Patients with negative [^{18}F] FCH PET/CT but increase in serum level of iPTH or calcium on clinical follow-up.
